# Retraction: Disruption of microRNA‐214 during general anaesthesia prevents brain injury and maintains mitochondrial fusion by promoting Mfn2 interaction with Pkm2

**DOI:** 10.1111/jcmm.18095

**Published:** 2023-12-28

## Abstract

Tiejun Liu, Bin Wang, Gai Li, Xiaoliu Dong, Guannan Yu, Qingzeng Qian, Likun Duan, Hongxia Li, Zhao Jia, Jing Bai. Disruption of microRNA‐214 during general anaesthesia prevents brain injury and maintains mitochondrial fusion by promoting Mfn2 interaction with Pkm2*. J Cell Mol Med*. 2020; 24: 13589–13599. https://doi.org/10.1111/jcmm.15222

The above article, published online on 04 November 2020 in Wiley Online Library (wileyonlinelibrary.com), has been retracted by agreement between the authors, the journal Editor‐in‐Chief, Stefan Constantinescu, The Foundation for Cellular and Molecular Medicine, and John Wiley and Sons Ltd. The retraction has been agreed following an investigation into concerns raised by a third party, which revealed inappropriate duplications of images between this and other articles that were previously published in a different scientific context. Thus, the editors consider the conclusions of this manuscript substantially compromised. Although initially the corresponding author contacted the editorial office and asked for retraction, the authors did not respond when asked to approve the wording.

Tiejun Liu, Bin Wang, Gai Li, Xiaoliu Dong, Guannan Yu, Qingzeng Qian, Likun Duan, Hongxia Li, Zhao Jia, Jing Bai. Disruption of microRNA‐214 during general anaesthesia prevents brain injury and maintains mitochondrial fusion by promoting Mfn2 interaction with Pkm2*. J Cell Mol Med*. 2020; 24: 13589–13599. https://doi.org/10.1111/jcmm.15222

The above article, published online on 04 November 2020 in Wiley Online Library (wileyonlinelibrary.com), has been retracted by agreement between the authors, the journal Editor‐in‐Chief, Stefan Constantinescu, The Foundation for Cellular and Molecular Medicine, and John Wiley and Sons Ltd. The retraction has been agreed following an investigation into concerns raised by a third party, which revealed inappropriate duplications of images between this and other articles that were previously published in a different scientific context. Thus, the editors consider the conclusions of this manuscript substantially compromised. Although initially the corresponding author contacted the editorial office and asked for retraction, the authors did not respond when asked to approve the wording.

